# To what extent do people in malaria-endemic countries know asymptomatic malaria infections? A systematic review

**DOI:** 10.1371/journal.pone.0340636

**Published:** 2026-01-16

**Authors:** Taofic Bouwe, Noudehouenou Credo Adelphe Ahissou, Kimiyo Kikuchi, Moritoshi Iwagami, Daisuke Nonaka

**Affiliations:** 1 Department of Global Health, Graduate School of Health Sciences, University of the Ryukyus, Ginowan, Okinawa, Japan; 2 SATREPS Project for Parasitic Diseases (JICA/AMED), Vientiane, Lao PDR; 3 Institute for Asian and Oceanian Studies, Kyushu University, Fukuoka, Japan; 4 Department of Global Health and Population, Harvard T.H. Chan School of Public Health, Boston, Massachusetts, United States of America; 5 Department of Tropical Medicine and Malaria, National Institute of Global Health and Medicine, Japan Institute for Health Security (JIHS), Tokyo, Japan; 6 Institut Pasteur du Laos, Ministry of Health, Vientiane, Lao PDR; University of Gondar College of Medicine and Health Sciences, ETHIOPIA

## Abstract

**Introduction:**

Malaria is a parasitic disease caused by *Plasmodium* parasite and transmitted to humans by infected female *Anopheles* mosquito. Several studies have explored community knowledge of malaria. However, despite the remarkable proportions of asymptomatic malaria, its awareness remains relatively understudied among the affected communities. Hence, this study aimed to determine the proportion of people with knowledge of asymptomatic malaria in endemic countries and describe their perspective towards malaria control interventions. The findings from this study could contribute to developing tailored interventions in both low and high-transmission settings.

**Method:**

The systematic review protocol was deposited in protocols.io and registered at PROSPERO (ID: CRD42024508104). A systematic literature review was conducted searching for peer-reviewed articles on knowledge of asymptomatic malaria published from 2010 to 2024. Three databases (PubMed (MEDLINE), Google Scholar, and Web of Science) were searched. The risk of bias in the included studies was assessed using the Joanna Briggs Institute (JBI) critical appraisal tool and the Mixed Methods Appraisal Tool (MMAT). A thematic analysis and a narrative synthesis were conducted to synthesise the results. The research followed the Preferred Reporting Items for Systematic Reviews and Meta-Analyses (PRISMA).

**Results:**

In total, 483 articles were retrieved, and 11 relevant articles were included in the analysis. According to four studies conducted among the general public, the proportion of individuals knowledgeable of asymptomatic malaria ranged from 14.2% to 79.8%. The proportion among health personnel was 88% (one study). The qualitative studies showed varied and lacking knowledge of asymptomatic malaria among the participants, as well as refusal and reluctance to adhere to interventions targeting asymptomatic *Plasmodium* carriers.

**Conclusion:**

This review showed a lack of knowledge of asymptomatic malaria among endemic communities and a remarkable shortage of studies on related topics. For better malaria control and to accelerate disease elimination, education on asymptomatic malaria would be necessary. Given the limited number of studies, further research on knowledge of asymptomatic malaria would be crucial in various malaria-endemic areas to provide evidence for tailored interventions.

## Introduction

Malaria is an acute febrile disease caused by a protozoan parasite (*Plasmodium*) which is transmitted to humans through a bite of an infected female *Anopheles* mosquito [1, 2]. It is the leading cause of death, particularly among children under 5 in endemic countries. Malaria is endemic in tropical and subtropical regions such as Central and South America, South and Southeast Asia, Oceania, and Sub-Saharan Africa, with the latter bearing the highest burden of the disease. Globally, the World Health Organisation (WHO) in 2023 estimated malaria deaths at 597,000 out of 263 million cases. The number of malaria deaths decreased by 4% from 2020, with a notable increase of 11 million cases from 2022 [3]. The most common *Plasmodium* species are *Plasmodium vivax* and *Plasmodium falciparum,* with the latter being the severest and deadliest due to the high parasite density in red blood cells during the infection and parasite sequestration into the blood vessels, leading to cerebral malaria among children in particular [4].

In the fight against malaria, control measures mainly focus on symptomatic infections (severe and mild malaria) with available point-of-care diagnostic tools, treatment, and protection tools [5]. Individuals with malaria symptoms such as headache, chills, fever, and vomiting will likely be aware of their condition and seek treatment at healthcare facilities. While several studies have explored the community’s knowledge of malaria, their focus was on people’s awareness of the disease’s symptoms, their attitude towards treatment seeking, and the protective measures they use [6–8]. On the other hand, asymptomatic malaria (AsM), defined as *Plasmodium* infection with any density without signs and symptoms related to the disease, constitutes one of the challenges to malaria control and elimination [9, 10]. A growing number of individuals in malaria-endemic settings develop very low levels of parasitemia with no visible symptoms. Or perhaps the widespread use of highly sensitive diagnostic methods, such as DNA diagnostic methods, has made it possible to reveal the burden of AsM or low-density *Plasmodium* infections. Although determining the burden or figures of AsM globally is challenging due to underreporting and diagnostic limitations, various studies in different countries reported relatively high prevalence of AsM [11–17]. Asymptomatic individuals are often diagnosed with comorbidities such as anemia, placental malaria, thrombocytopenia, and cognitive impairment, mostly in children [18].

Moreover, individuals with asymptomatic infections constitute malaria reservoirs and may contribute to disease transmission because mosquitoes feeding on blood from them can become infected and perpetuate malaria transmission [19].

Despite the increasing proportions of AsM, its implication on health, and its impediment to malaria control and elimination, awareness of asymptomatic *Plasmodium* infections remains relatively understudied among the affected communities [20]. To our knowledge, studies that have reported community knowledge of AsM are limited in number [21–31], and reviews on the related topic are almost non-existent. It is therefore crucial to explore and raise awareness on AsM as this knowledge may determine the community level of engagement in malaria control interventions such as Intermittent Preventive Treatment, Seasonal Malaria Chemoprevention, Mass Drug Administration, Targeted Testing and Treatment, and Reactive Case Detection and Treatment [3].

Hence, this systematic review aims to determine the proportion of people with knowledge of AsM in endemic countries and describe their perspectives towards malaria control interventions. This literature will contribute to evidence-based public health strategy development for targeted interventions.

## Method

### Registration

This systematic review protocol was deposited in protocols.io and was registered at PROSPERO (ID: CRD42024508104). The report was conducted following the Preferred Reporting Items for Systematic Reviews and Meta-Analyses (PRISMA) statement for reporting systematic reviews, “S2 File”.

### Eligibility criteria

Peer-reviewed English-language articles on knowledge, perceptions, and awareness of AsM published between 1^st^ January 2010 and 26^th^ May 2024 were sought to be included, while studies on simian malaria other than *Plasmodium knowlesi,* case reports, opinion papers, and editorials were excluded. The included studies were restricted to publications from 2010 to 2024 due to the shift in global malaria control and elimination strategies [32–34]. Although asymptomatic infections have long been recognized as contributors to transmission, their systematic targeting through active case detection (ACD) has become a central focus in global and national malaria elimination efforts since early 2010 [35]. These strategies would have led to increased research attention and funding directed towards understanding and addressing asymptomatic malaria. Hence, this time frame may capture broader literature relevant to our topic.

Searched articles comprised cross-sectional, cohort, mixed-method, baseline surveys, intervention, and qualitative studies.

### Search strategy

Searches in the following databases, PubMed (MEDLINE), Google Scholar, and Web of Science, were conducted from 30/03/2024 to 26/05/2024. The search strategies were as follows: PubMed (MEDLINE): (((community) AND (attitude OR knowledge OR awareness OR perception)) AND (asymptomatic OR afebrile OR symptomless)) AND (malaria); Google Scholar: attitude knowledge awareness perception afebrile OR symptomless OR community “asymptomatic malaria”; Although Google Scholar includes grey literature, we sorted results by relevance and all non–peer-reviewed items and grey literature were removed during the title and abstract screening stage. Web of Science: (((ALL=(community)) AND ALL=((knowledge OR awareness OR perception OR attitude))) AND ALL=(asymptomatic OR afebrile OR symptomless)) AND ALL=(malaria). The search string differed in each database because we considered the keywords and operators that yielded more articles. In addition to the database search, we conducted citation search until October 2024 to identify further relevant studies, “S3 File”.

### Screening strategy

The initial screening stages were done using Zotero software (Zotero, Version 6.0.36, Corporation for Digital Scholarship, 2024) to remove duplicates. Then, to identify studies that potentially met the inclusion criteria, two authors, TB and NCAA, independently screened the titles and abstracts of the studies. The full texts of the eligible studies were retrieved and independently assessed by TB and NCAA. Disagreements regarding the eligibility of particular studies were resolved through discussion with a third author (DN).

### Data extraction

For data extraction, a standard form (spreadsheet) was used to extract the following information from eligible articles: the study year and publication year, study design, study sites, participants’ age group, and study outcomes. These data were extracted independently by TB and NCAA. A third author (DN) was consulted to resolve discrepancies when necessary.

### Outcomes

The primary outcome is the proportion of individuals who know AsM. The secondary outcome is the community’s perspective on interventions targeting asymptomatic individuals.

### Risk of bias assessment

The risk of bias in the included studies was assessed using the Joanna Briggs Institute (JBI) critical appraisal tool [36]. The critical appraisal tool checklists specific to each study design, such as cross-sectional and qualitative studies, were utilized. The Mixed Methods Appraisal Tool (MMAT) was employed for mixed methods studies [37]. The appraisal questions were answered with yes, no, or unclear. Studies that met most or all the criteria “yes” were classified as “high quality”, those that met the majority of the criteria “yes” and had some “no” and/or “unclear” were classified as “moderate quality” and the studies with most “no” and/or “unclear” were classified as “low quality” “S4 File”.

### Data synthesis

The proportions of participants with knowledge of AsM were retrieved to determine the pooled proportion in STATA software version 14 using a random effect model. The heterogeneity among studies was determined by I^2^ statistics. However, due to the limited number of quantitative studies (only five), further analyses, such as subgroup and sensitivity analyses, could not be performed. Consequently, meta-analysis was deemed unsuitable [38]. Therefore, for quantitative studies (cross-sectional and mixed methods), the median of the proportions of individuals with knowledge of AsM was determined to show the central tendency, and a narrative synthesis was performed using descriptive statistical analysis [39]. For qualitative studies, a thematic analysis was conducted, followed by narrative synthesis.

### Definition of “knowledge of asymptomatic malaria”

In the included articles, participants’ knowledge of AsM was determined primarily by asking yes or no questions such as “Have you ever heard about asymptomatic malaria?” or “Can someone have malaria without showing symptoms?” etc. Hence, for operationalization in this systematic review, knowledge of asymptomatic malaria (AsM) is defined as “People’s awareness that malaria infection can occur without symptoms”. Nevertheless, it is worth noting that some studies explored participants’ understanding, perception, and attitude towards AsM.

## Results

### Search results

**Fig 1:**
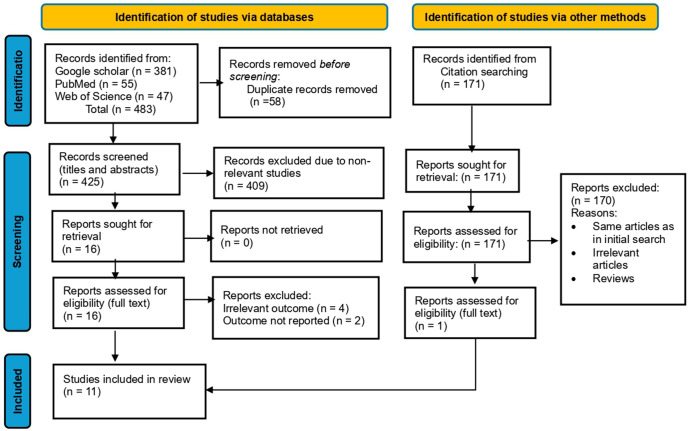
PRISMA flow diagram illustrating the study selection process.

In total, 483 articles were retrieved from database searches (381 from Google Scholar, 55 from PubMed, and 47 from Web of Science. After removing 58 duplicate records, 425 articles were screened for title and abstract. After screening, 409 were excluded for non-relevance. Thereafter, 16 relevant articles were included in the full-text examination, from which six articles were excluded due to irrelevant outcomes (4 articles) and outcomes not reported (2 articles) [40, 41]. The additional search from citations retrieved 171 articles from which only one article was included in the review [31]; the remaining articles from the citation search were excluded for the following reasons: same as included articles in the initial search, non-relevant, and reviews. The additional article included did not change the findings but enhanced the comprehensiveness of the review. Hence, 11 articles in total were included in the systematic review “Fig 1. PRISMA flow diagram illustrating the study selection process”.

### Characteristics of the included studies

The detailed features of each article included are in Table 1. Eight studies (72.7%) were from Africa: Kenya (2), Mozambique (1), Eswatini (1), Cameroon (1), Ghana (1), Gambia (1), and Senegal (1). The remaining three articles (27.3%) were from Southeast Asia: Laos (2) and Cambodia (1). Five papers were qualitative studies, two were quantitative, and four were mixed methods. Most of the studies included participants from the general public; one study from Cameroon involved only health personnel, and two studies from Senegal and Mozambique included participants from the general public and health personnel. The ages of the participants ranged from 12 to 80 years old [21–31].

**Table 1:** Summary of the studies included.

	Authors	Study year	Publication year	Country	Study design	Participants	Age range (yrs)	Quality score**
1	**Cuinhane, Carlos Eduardo et al.**	2022	2023	Mozambique	Qualitative	n = 239	≥12	High
2	**Adhikari, Bipin et al.**	2015-2016	2018	Laos	Qualitative	n = 131	18–80	High
3	**Baltzell, Kimberly A. et al.**	2015-2017	2019	Eswatini	Qualitative	n = 208	18–80	High
4	**Okello, George, et al.**	2010-2012	2012	Kenya	Qualitative	NA	adults	High
5	**Shuford, Kathryn, et al.**	2013	2016	Kenya	Qualitative	n = 334	adults	High
**Quantitative Studies**								
6	**Nchang, Abenwie Suhet al.**	2022	2023	Cameroon	Cross-sectional	n = 100	18–65	Moderate
7	**Orish, Verner N et al.**	2022	2023	Ghana	Cross-sectional	n = 200	≥15	Moderate
**Mixed Methods**								
8	**Adhikari, Bipin et al.**	2015	2018	Laos	Cross-sectional/ quali	n = 281	≥18	High
9	**Jaiteh, Fatou, et al.**	2016	2019	Gambia	Cross-sectional/ quali	Quanti *: n = 741 Quali *: n = 93	≤16 & adults	High
10	**Peto, Thomas J. et al.**	2015-2016-2017	2018	Cambodia	RCT/ Quali	Quanti n = 163 Quali: NA	≥18	High
11	**Legendre et al.**	2021-2022	2024	Senegal	cross-sectional/ quali.	Quanti n = 289 Quali n = 120	≥15	High

*: Quant = quantitative; Quali = qualitative; RCT = Randomised Control Trial.

**: See supplementary file “S4 File”.

### Quantitative results

#### Heterogeneity among studies.

Out of 11 studies included, only five quantitative studies provided enough data for proportion estimation. The meta-analysis using a random effect model showed I^2^ = 99.4%, p-value < 0.001, indicating considerable heterogeneity. Despite the remarkable variability among studies, that is, clinical heterogeneity (differences in participants) and methodological heterogeneity (different study designs), we did not conduct subgroup and sensitivity analyses due to the very small number of studies [38]. Therefore, meta-analysis results were not considered. We proceeded with a narrative synthesis and calculated the median to show the central tendency of the proportion of individuals with knowledge of AsM.

#### Proportion of individuals knowledgeable of asymptomatic malaria.

Four studies (two quantitative and two mixed methods) conducted among the general public reported the proportion of people knowledgeable of AsM, which ranged from 14.2% to 79.8% with a median of 66% (Interquartile range: 47% to 75%). One quantitative study conducted among healthcare personnel (physicians, nurses, and nursing assistants) reported 88% of knowledge of AsM [26]. The total number of participants in the quantitative studies (including quantitative strands of mixed methods) was 1,774. The total number of participants in the qualitative studies could not be determined as not all studies reported the number of participants. The average age of participants could not be determined as some studies did not report the lower or upper age limit of participants.

In the study conducted in Ghana, 58% of individuals were knowledgeable of AsM [27]. In the Gambia, this proportion was 73% [29], while in Cambodia it was 79.8% [30]. The study conducted in Lao PDR reported that 14.2% of individuals had knowledge of symptomless malaria “Table 2”, while a third (33.8%) responded that they did not know, and over half (52%) rejected this idea [28].

**Table 2:** Proportion of participants with knowledge of AsM.

	Author & year	Country	Study design	participants Category	Sample size	Proportion of Knowledge of AsM (%)	95% CI*
1	Nchang, Abenwie Suhet al. (2022)	Cameroon	Cross-sectional	Health personnel	100	88	0.8 to 0.9
2	Orish, Verner N et al. (2022)	Ghana	Cross-sectional	Urban population	200	58	0.5 to 0.6
3	Adhikari, Bipin et al. (2015)	Lao PDR	Mixed methods	Rural community	281	14.2	0.1 to 0.2
4	Jaiteh, Fatou et al. (2016)	Gambia	Mixed methods	Rural community	741	73	0.7 to 0.8
5	Peto, Thomas J. et al. (2015)	Cambodia	Mixed methods	Rural community	163	79.8	0.7 to 0.9

NB: 95% CI* values were calculated from the confidence interval for proportion.

#### Trend of asymptomatic malaria knowledge.

In the studies conducted in the Gambia and Lao PDR [26, 28, 29], there was no remarkable difference in age groups, gender, and knowledge of AsM. The same observation was made among healthcare workers in Cameroon, but nursing assistants had a lower Knowledge of AsM [26]. In the study conducted in Ghana where knowledge of AsM was scored based on different information (signs, symptoms, transmission and treatment) [27], higher knowledge of AsM was observed among those aged 21–30 yrs (p-value < 0.001) and 31–40 yrs (p-value = 0.014) compared to those above 50 yrs; individuals with tertiary education were more knowledgeable about AsM than senior and junior high schoolers (both p-value < 0.001); government employees had also higher knowledge of AsM compared to private sector workers (p-value = 0.001). More males (31.9%) than females (19.8%) had high knowledge about AsM; For moderate knowledge, females accounted for 59.3% and males 44.5%. All the p-values are from the original articles.

#### Understanding, attitudes, and perceptions of asymptomatic malaria.

Although data are sparse and lacking across studies, some studies provided in-depth information on knowledge of AsM.

**Understanding**: this is a deep knowledge that refers to factual awareness or accurate information that individuals have on AsM (i.e., its existence, diagnosis, treatment, and transmission)

In Cameroon, 75% (66/88) of the healthcare workers, as well as 86.5% of participants in Ghana who knew of AsM, reported that it could be diagnosed. Also, participants in Cameroon (51.1% of healthcare workers), Ghana (33.5%), and Lao PDR (36.7%) reported that asymptomatic infections could perpetuate malaria [26–28] “S5 File”.

**Attitude**: Participants’ attitudes are their behavioural intentions towards testing, treating, or learning more about asymptomatic malaria.

In Cameroon, 85.2% (75 out of 88) of healthcare workers reported having encountered asymptomatic malaria (AsM), and among them, 93% (70 out of 75) provided treatment for the condition [26]. While in Ghana, 76% of the participants reported being willing to take a malaria test without symptoms, and 90% reported they would accept treatment if tested positive but asymptomatic; at the same time, 92% were willing to receive education on AsM. In contrast, the study in Senegal reported that 41.5% of the participants were reluctant to malaria treatment without symptoms [31].

**Perception** These results show how individuals emotionally or subjectively viewed AsM.

The study in the Gambia reported that 71% of the participants claimed that there is an increased risk of infection while living in the same household with a malaria-infected person. In the study conducted in Ghana, 67% of the participants thought AsM is a serious health condition, and 53% feared having it.

#### Qualitative results.

Different types of interviews, namely “in-depth interviews”, “focus group discussion”, “semi-structured interviews”, and “informal conversations” were conducted, “S5 File”. Three key themes evolved from the qualitative data analysis: Knowledge (understanding or awareness), Malaria test and drug administration without symptoms (attitude and perception), and Sensitization on asymptomatic malaria (AsM) “S6 File.

1 **Knowledge-Understanding-Perception of asymptomatic malaria**

In this theme, different levels of awareness and understanding from the participants are highlighted. In studies carried out in Lao PDR, Gambia, Eswatini, and Kenya, the answers from interviews range from misconceptions to basic accurate knowledge [22–25, 29].

**Health personnel’s Understanding of asymptomatic malaria**

Healthcare personnel (health manager) showed a deep understanding of AsM and its treatment implications.

*“I think this one is quite focused because you have actually tested someone and seen parasites so you know exactly what you are dealing with. And you see this will also have the potential impact of removing the hosts because you see those with the parasites will not have the symptoms...so you are reducing transmission as well.”* (Health manager, IDI) [24].

This highlights the difference in knowledge between healthcare professionals and the general public.

**Community understanding of asymptomatic malaria**

Some participants were aware that AsM can only be detected through testing, showing a basic but appropriate understanding of its asymptomatic nature. Asymptomatic individuals were also recognized by a few participants as having the potential to perpetuate malaria transmission, which reflects an understanding of the public health implications of AsM.

*“Someone can live with malaria in their bodies for long without knowing it…you think you don’t have malaria but it’s already in you, so if you get an opportunity to be tested before the malaria symptoms show in your body then that will be a good opportunity.” (Female FGD, Asembo)*[25].

**Perception of asymptomatic malaria**

Some participants linked AsM infections to personal or surrounding tidiness or their occupation. This shows a misconception between asymptomatic *Plasmodium* infection and the risk of malaria transmission. Some parents viewed AsM as a less concerning health issue, especially for school children who they think may recover on their own. This perception misjudges the threat of asymptomatic malaria infections, mainly the potential spread of malaria.

*“Do you think that a healthy person can have malaria parasites in his/her body? For example, me, do you think I have malaria parasite in my body or not/ No, because you look clean. You do not have to go to forest like us. For us, we have to go to forest every day to find food. We do not have good clothes. I just only have one blanket in my house, I have to give it to my husband and my kids.” 30-year-old female Focus Group Discussion participant,*[22].

Some participants believed that asymptomatic infections constitute an early stage of malaria, and symptoms may appear later following the disease progression. *“It can happen as you can find that it is still in its early stages…” Tha bankulu FGD participant)*[23].

2 **Community attitude towards asymptomatic malaria (testing and drug administration)**

In this theme, participants’ opinions varied on malaria testing and drug administration without symptoms in studies conducted in Kenya, Eswatini, Lao PDR, Mozambique, and Senegal.

*“From my experience I cannot accept to be tested when I don’t feel sick…I will wait until I feel sick to be tested.” (Opinion leader FGD, Asembo)*[25].

Conversely, some participants accepted taking antimalarial drug while they were healthy without a deep understanding of AsM. However, they believed taking antimalarial drug reduces disease cases.

*“I accept taking tablets even without malaria. Even if field workers leave my neighbor’s house after giving pills…” (SSI 09, community leader, Magude village)*[21].

3 **Sensitization on the Rationale of Asymptomatic Malaria**

In studies conducted in Mozambique, Kenya, and Senegal, participants being aware of their lack of information and knowledge, responded by asking for education and sensitization regarding AsM.

*“It requires sensitization and making people understand that there are asymptomatic carriers who are present and transmit the disease to others without knowing. Now, we need to sensitize them to tell them the importance of the project.’‘- Man, FG.*[31].

## Discussion

This review provided a summary of qualitative and quantitative data on communities’ knowledge about AsM in endemic settings and their attitude and perceptions of interventions targeting AsM carriers. The key findings in this study are the proportion of individuals knowledgeable of AsM (median: 66%), the lack of awareness of AsM implications, the varied perceptions of interventions targeting asymptomatic individuals, and the lack of studies on knowledge of AsM in malaria-endemic communities.

### Proportion of individuals with knowledge of asymptomatic malaria

The quantitative results showed that the proportion of the general public’s knowledge of asymptomatic *Plasmodium* infection varied in different countries and among participants, with a range of 14.2% to 79.8%; this knowledge was 88% among health personnel. The knowledge and awareness were low in Lao PDR (14.2%), moderate in Ghana (58%), and high in the Gambia (73%), Cambodia (79.8%), and Cameroon (88% with health personnel). The difference in the proportion of individuals knowledgeable of AsM could be related to participants’ educational level, occupation, and/or the presence of health programmes on malaria awareness in the study areas [42–44]. As shown here, the highest proportion was found in Cameroon among health personnel. For health programmes on malaria, for instance in Cambodia, it was reported that participants probably attended some meetings where staff explained the study and the concept of AsM prior to the survey, which would have increased people’s awareness [30]. Additionally, in the study conducted in the Gambia, some health promotion activities explained to the participants the process of natural immunity against parasites [29]. This also might contribute to the insights gained about AsM.

### Demographic factors and trends in knowledge of asymptomatic malaria

In studies from Cameroon, Gambia, and Lao PDR, age and gender were not substantially associated with knowledge of AsM. However, in Ghana, adults (21–40 yrs, p-value < 0.05) and males, government employees (p-value = 0.001), and those who had tertiary education (p-value < 0.001) had higher knowledge of AsM, although the perception score did not differ among these variables. This may be related to their exposure to health education programmes and their literacy, which may influence their capacity to understand health-related problems. Furthermore, government employees and adults may have more access to health information through their workplace health plans or seek health information more actively due to the responsibilities of caring for family [45–48]. These findings corroborate studies from Malaysia, Zanzibar, and a systematic review from Sub-Saharan Africa [49–51].

Education and occupation remarkably influenced knowledge about AsM. Higher-trained health personnel (physicians, nurses) tend to have a better understanding of AsM, although this understanding was low among assistant nurses in Cameroon. The low knowledge among assistant nurses could be explained by the difference in their training and specific tasks. This is in line with studies on cognitive dysfunction and sickle cell disease, where differences in knowledge were reported among physicians and nurses, denoting that training plays an important role in developing disease-specific knowledge [52, 53].

### Understanding of asymptomatic malaria

Different proportions of participants who understood symptomless malaria patterns, such as its diagnosis and its potential to perpetuate malaria, were reported. The substantial proportion of healthcare professionals in Cameroon (75%) who reported that AsM can be diagnosed shows good awareness and understanding. This could enable better participation in the AsM case detection strategies and patient sensitization. However, it also highlights a non-negligible proportion (25%) that lack an appropriate understanding of AsM. This may be concerning since healthcare personnel are implementers of malaria diagnosis and treatment. The lack of full knowledge of AsM among certain personnel may reflect a lack of knowledge update or insufficient training.

In Ghana, with the general public, 86.5% of the participants responded that getting a malaria test allows them to reveal asymptomatic infections. This considerable proportion of people aware of AsM diagnosis could be related to participants’ experience in seeking treatment at healthcare facilities for symptomatic infections. This awareness is important for their adherence to interventions such as mass screening and treatment. Nonetheless, it is crucial to note that this percentage of people with such awareness should not be representative of the country, as the sample size was small (200), and only a few participants with lower educational backgrounds joined the study.

Moreover, 51%, 33.5%, and 36.7% of participants in Cameroon, Ghana, and Lao PDR, respectively, reported that symptomless *Plasmodium* carriers could perpetuate the disease. This implies that although some participants are well aware of asymptomatic infections’ implications, a large number of them do not grasp their threat, which may impede malaria control efforts, specifically those targeting asymptomatic individuals.

### Attitude towards asymptomatic malaria

In Cameroon, a notable proportion of healthcare professionals (93%) who had encountered AsM provided treatment. This finding shows that the majority of health professionals were aware of AsM implications and managed symptomless malaria cases appropriately. This approach of treating AsM is in line with a study advocating the treatment of all malaria cases [9]. On the other hand, only a few personnel did not provide treatment for AsM cases, which may imply either a different interpretation of treatment guidelines or a lack of awareness of AsM implications.

Furthermore, some of the study participants showed willingness, and others showed hesitancy in testing for malaria and taking antimalarial drugs without symptoms. A considerable proportion of individuals (76%, Ghana) exhibited a willingness to test for malaria even without symptoms, with a substantial proportion of them willing to take treatment if tested positive [27]. Conversely, 41.5% of participants in Senegal showed reluctance to malaria treatment in the absence of symptoms [31]. These results are substantiated by varied opinions in the qualitative component. Some participants in the qualitative studies refused testing or taking drugs without malaria symptoms, while others viewed it as an opportunity to protect themselves against any potential disease [21, 23–25].

The notable reluctance to get tested or take antimalarial medication without symptoms may pose a significant barrier to proactive malaria control (specifically for asymptomatic individuals). The belief that treatment should only follow a positive test or the presence of symptoms can disrupt efforts to address asymptomatic *Plasmodium* infections. This suggests the necessity of explaining the rationale behind interventions such as reactive malaria testing, MDA, or TDA [22]. In addition, public health strategies need to tackle these beliefs through education on the importance of early testing and the role of asymptomatic individuals in spreading the disease.

### Request for education on asymptomatic malaria

Although many participants lacked knowledge of AsM, some of them recognized the importance of education about asymptomatic infections. In the study by Orish, Verner N et al., 92% of the participants expressed their will to get education on malaria [27]. Similarly, some respondents in the qualitative studies clearly demanded sensitization on symptomless malaria. This suggests some participants are aware of their lack of health-related knowledge. These gaps may appropriately be addressed by health interventions focusing on the implications of asymptomatic carriers and the importance of early detection and treatment.

### Perceptions

Participants perceived differently AsM. In the quantitative findings, a good proportion of the community (71%, Gambia) stated that living in the same household with a malaria-infected person increases the risk of infection. On one hand, this may suggest a misconception of the transmission mode as malaria is not transmitted directly from person to person. On the other hand, the statement may indicate awareness of how asymptomatic individuals, serving as reservoirs, can spread the disease through mosquitoes.

Additionally, the misconception of AsM was also revealed in the qualitative results, as some respondents referred to AsM as merely an early stage of malaria, and symptoms would appear later. This perception may lead to nonadherence to interventions such as targeted drug administration (TDA) or mass drug administration (MDA), as people would be waiting to act only when symptoms appear [28].

The knowledge, understanding, attitudes, and perceptions of AsM discussed above may be context-specific, as the regional variations in malaria transmission intensity, population immunity, and healthcare infrastructure may influence awareness and comprehension of symptomless malaria [54].

### Limitations of the study

This study has some limitations. The meta-analysis results were not considered due to the limited number of quantitative studies. Additionally, the included studies did not report all necessary data on the community knowledge of AsM. Furthermore, the limited geographical coverage of the studies (Africa and Asia) restricts the generalizability of the findings to other malaria-endemic regions, namely the Western Pacific and South America. As such, our inferences might not thoroughly represent AsM knowledge in regions not covered by the studies we included. Nevertheless, the findings gathered in this review still provide relevant information for areas with a similar social and epidemiological context, given the fact that Africa and Asia bear the greatest malaria load globally.

## Conclusion

This review showed a lack of knowledge of AsM among endemic communities and a remarkable shortage of studies on related topics. Although some participants expressed their willingness to get malaria tests and/or take antimalarial drugs while asymptomatic, others abide by the idea that one should test malaria positive or have symptoms before treatment.

### Recommendation

To bridge knowledge gaps, interventions tailored to each endemic setting should integrate AsM content into health education programmes and malaria prevention campaigns. Furthermore, given the scarcity of studies, more research on knowledge of AsM, both quantitative and qualitative, is needed.

## Supporting information

S1 File Prospero protocol.pdf. (PDF)

S2 File PRISMA checklist. (ZIP)

S3 File Database search and strategies. (DOCX)

S4 File Critical appraisal. (XLSX)

S5 File Details of included studies. (XLSX)

S6 File Qualitative data. (XLSX)

## Abbreviations

AsM: Asymptomatic Malaria
FGD: Focus Group Discussion
IDI: In-Depth Interview
JBI: Joanna Briggs Institute
Lao PDR: Lao People’s Democratic Republic
MDA: Mass Drug Administration
MMAT: Mixed Methods Appraisal Tool
PRISMA: Preferred Reporting Items for Systematic Review and Meta-analysis
PROSPERO: International Prospective Register of Systematic Reviews
SSI: Semi-Structured Interview
TDA: Targeted Drug Administration.
