# Supplementary material for: To what extent do people in malaria-endemic countries know asymptomatic malaria infections? A systematic review
Source: PLoS One. 2026 Jan 16;21(1):e0340636. doi: 10.1371/journal.pone.0340636 (PMC12810798; doi:10.1371/journal.pone.0340636)
Supplement: S1 File — (PDF) [file pone.0340636.s001.pdf]

To what extent do people in malaria-endemic countries know asymptomatic malaria infections? A Systematic Review.

To enable PROSPERO to focus on COVID-19 submissions, this registration record has undergone basic automated checks for eligibility and is published exactly as submitted. PROSPERO has never provided peer review, and usual checking by the PROSPERO team does not endorse content. Therefore, automatically published records should be treated as any other PROSPERO registration. Further detail is provided here.

Citation

Taofic Bouwe, Noudéhouénou Crédo Adelph Ahissou, Kimiyo Kikuchi, Moritoshi Iwagami, Daisuke Nonaka. To what extent do people in malaria-endemic countries know asymptomatic malaria infections? A Systematic Review.. PROSPERO 2024 CRD42024508104 Available from:
https://www.crd.york.ac.uk/prospero/display_record.php?ID=CRD42024508104

Review question

To what extent do people in malaria-endemic countries know asymptomatic malaria infections?

Searches

The search will be in English language with a strategy that includes both subject headings and text words relating to or describing the topic (community, knowledge, asymptomatic malaria).

Search terms : "community" "awareness" "knowledge" "attitude" and "asymptomatic malaria".

Databases : PubMed (MEDLINE), Google Scholar, EMBASE, Emcare, Global Health and Web of Science.

References and citations of the articles included in the review will be searched for additional relevant studies.

Types of study to be included

Quantitative studies:

- Cross-sectional, cohort, case-control and intervention studies

Qualitative studies

- In-depth interviews, focus group interviews, focus group discussions.

Condition or domain being studied

Asymptomatic malaria: malaria infection with plasmodium parasites of any density without symptoms related to the disease.

Participants/population

Studies involving individuals or communities residing in malaria-endemic countries.

Intervention(s), exposure(s)

Not applicable

Comparator(s)/control

Not applicable

Context

Asymptomatic malaria in malaria-endemic countries

Main outcome(s)

Proportion of the Population who know about asymptomatic malaria

Additional outcome(s)

Awareness, perceptions, or beliefs about asymptomatic malaria

Data extraction (selection and coding)

To identify studies that potentially meet the inclusion criteria, two authors will independently screen titles and abstracts of studies retrieved using the search strategy as well as those from additional sources. The full text of these eligible studies will be retrieved and independently assessed by two review team members. Disagreements between them regarding the eligibility of particular studies will be resolved through discussion with a third author.

A standard form will be used to extract the following information: the year when the study was conducted, year of study publication, study sites, study population and age groups, and baseline characteristics, study design, outcomes, information for risk of bias assessment. Data will be extracted independently by two authors. When necessary, a third author will be consulted to resolve inconsistancies.

Risk of bias (quality) assessment

For the quality assessment of included studies, the Joanna Briggs Institute (JBI) critical appraisal tool will be used. specific critical appraisal checklist tools dedicated to each study design will be applied. For cross-sectional studies, a checklist of 8 criteria will be applied, covering aspects such as the clarity of inclusion criteria, identification of confounding factors and the validity and reliability of outcome measurements. A checklist of 10 criteria will be applied to case-control studies (e.g. Were confounding factors identified?), while for cohort studies, a checklist tool with 11 criteria will be applied, including the following aspects: group similarity and recruitment from the same population. Intervention studies will be subject to a checklist of 9 criteria covering aspects such as the existence of multiple outcomes both before and after the intervention, and qualitative studies will be evaluated using a 10-criteria checklist, covering aspects like congruity between research methodology and research questions or objectives, as well as the interpretation of results.

Strategy for data synthesis

For quantitative studies, the proportions of participants aware of asymptomatic malaria and confidence intervals from the included studies will be pooled. Due to heterogeneity among included studies (i.e. various study designs), the summary of the effect size will be conducted using a meta-analysis random-effects model. For qualitative studies, we will descriptively summarize findings on Awareness, perceptions or beliefs about asymptomatic malaria and provide a narrative synthesis.

Analysis of subgroups or subsets

Statistical heterogeneity will be assessed using I^2 Statistic and if necessary, a sub-group analysis will be conducted to explore heterogeneity among and between studies for a better explanation of the observed differences.

Contact details for further information

TAOFIC BOUWE

btaofic97@gmail.com

Organisational affiliation of the review

Department of Global Health, Graduate School of Health Sciences, University of the Ryukyus, Okinawa, Japan

https://www.med.u-ryukyu.ac.jp/

Review team members and their organisational affiliations

Mr Taofic Bouwe. Department of Global Health, Graduate School of Health Sciences, University of the Ryukyus, Okinawa, Japan

Mr Noudéhouénou Crédo Adelph Ahissou. Department of Global Health, Graduate School of Health Sciences, University of the Ryukyus, Okinawa, Japan

Dr Kimiyo Kikuchi. Office of Academic International Affairs, Graduate School of Medicine and Faculty of Medicine, The University of Tokyo, Japan / Department of Global Health and Population, Harvard T.H. Chan School of Public Health, USA

Dr Moritoshi Iwagami. Department of Tropical Medicine and Malaria, Research Institute, National Center for Global Health and Medicine (NCGM), Japan

Dr Daisuke Nonaka. Department of Global Health, Graduate School of Health Sciences, Faculty of Medicine, University of the Ryukyus, Okinawa, Japan

Type and method of review

Meta-analysis, Systematic review

Anticipated or actual start date

30 March 2024

Anticipated completion date

30 December 2024

Funding sources/sponsors

Japan Agency for Medical Research and Development for "Project for malaria and neglected parasitic diseases control and elimination using advanced research technique, communication tools and eco-health education" under International Collaborative Research Program: Science and Technology Research Partnership for Sustainable Development (SATREPS)

Conflicts of interest

Language

English

Country

Japan

Stage of review

Review Ongoing

Subject index terms status

Subject indexing assigned by CRD

Subject index terms

MeSH headings have not been applied to this record

Date of registration in PROSPERO

12 February 2024

Date of first submission

01 February 2024

Stage of review at time of this submission

The review has not started

Stage	Started	Completed
Preliminary searches	No	No
Piloting of the study selection process	No	No
Formal screening of search results against eligibility criteria	No	No
Data extraction	No	No
Risk of bias (quality) assessment	No	No
Data analysis	No	No

The record owner confirms that the information they have supplied for this submission is accurate and complete and they understand that deliberate provision of inaccurate information or omission of data may be construed as scientific misconduct.

The record owner confirms that they will update the status of the review when it is completed and will add publication

details in due course.

Versions

12 February 2024

12 February 2024
