# Supplementary material for: To what extent do people in malaria-endemic countries know asymptomatic malaria infections? A systematic review
Source: PLoS One. 2026 Jan 16;21(1):e0340636. doi: 10.1371/journal.pone.0340636 (PMC12810798; doi:10.1371/journal.pone.0340636)
Supplement: S2 File — (ZIP) [file pone.0340636.s002.zip › S2B_PRISMA_2020_abstract_checklist.docx]

To what extent do people in malaria-endemic countries know asymptomatic malaria infections? : A systematic review

**S3**

| **Section and Topic** | **Item #** | **Checklist item** | **Reported (Yes/No)** |
| --- | --- | --- | --- |
| **TITLE** | | |  |
| Title | 1 | Identify the report as a systematic review. | 1 |
| **BACKGROUND** | | |  |
| Objectives | 2 | Provide an explicit statement of the main objective(s) or question(s) the review addresses. | 2 |
| **METHODS** | | |  |
| Eligibility criteria | 3 | Specify the inclusion and exclusion criteria for the review. | 2 |
| Information sources | 4 | Specify the information sources (e.g. databases, registers) used to identify studies and the date when each was last searched. | 2 |
| Risk of bias | 5 | Specify the methods used to assess risk of bias in the included studies. | 2 |
| Synthesis of results | 6 | Specify the methods used to present and synthesise results. | 2 |
| **RESULTS** | | |  |
| Included studies | 7 | Give the total number of included studies and participants and summarise relevant characteristics of studies. | 2 |
| Synthesis of results | 8 | Present results for main outcomes, preferably indicating the number of included studies and participants for each. If meta-analysis was done, report the summary estimate and confidence/credible interval. If comparing groups, indicate the direction of the effect (i.e. which group is favoured). | 2 |
| **DISCUSSION** | | |  |
| Limitations of evidence | 9 | Provide a brief summary of the limitations of the evidence included in the review (e.g. study risk of bias, inconsistency and imprecision). | 3 |
| Interpretation | 10 | Provide a general interpretation of the results and important implications. | 3 |
| **OTHER** | | |  |
| Funding | 11 | Specify the primary source of funding for the review. | 3 |
| Registration | 12 | Provide the register name and registration number. | 2 |

*From:*  Page MJ, McKenzie JE, Bossuyt PM, Boutron I, Hoffmann TC, Mulrow CD, et al. The PRISMA 2020 statement: an updated guideline for reporting systematic reviews. BMJ 2021;372:n71. doi: 10.1136/bmj.n71. This work is licensed under CC BY 4.0. To view a copy of this license, visit <https://creativecommons.org/licenses/by/4.0/>
