# Supplementary material for: To what extent do people in malaria-endemic countries know asymptomatic malaria infections? A systematic review
Source: PLoS One. 2026 Jan 16;21(1):e0340636. doi: 10.1371/journal.pone.0340636 (PMC12810798; doi:10.1371/journal.pone.0340636)
Supplement: S3 File — (DOCX) [file pone.0340636.s003.docx]

**S3_Databases and search strategies**

To what extent do people in malaria-endemic countries know asymptomatic malaria infections? : A systematic review

| Database & last date of search | Search terms | Search link | Number of hits |
| --- | --- | --- | --- |
| Google Scholar  26/05/2024 | attitude knowledge awareness perception afebrile OR symptomless OR community "asymptomatic malaria" | [attitude knowledge awareness perception afebrile... - Google Scholar](https://scholar.google.com/scholar?start=0&q=attitude+knowledge+awareness+perception+afebrile+OR+symptomless+OR+community+%22asymptomatic+malaria%22&hl=en&as_sdt=0,5&as_ylo=2010&as_yhi=2024) | 381 |
| PubMed  26/05/2024 | (((community) AND (attitude OR knowledge OR awareness OR perception)) AND (asymptomatic OR afebrile OR symptomless)) AND (malaria) | [(((community) AND (attitude OR knowledge OR awareness OR perception)) AND (asymptomatic OR afebrile OR symptomless)) AND (malaria) - Search Results - PubMed (nih.gov)](https://pubmed.ncbi.nlm.nih.gov/?term=(((community)%20AND%20(attitude%20OR%20knowledge%20OR%20awareness%20OR%20perception))%20AND%20(asymptomatic%20OR%20afebrile%20OR%20symptomless))%20AND%20(malaria)&sort=&page=3) | 55 |
| Web of Science  26/05/2024 | (((ALL=(community)) AND ALL=((knowledge OR awareness OR perception OR attitude))) AND ALL=(asymptomatic OR afebrile OR symptomless)) AND ALL=(malaria) | [(((ALL=(community)) AND ALL=((knowledge OR awareness OR perception OR attitude))) AND ALL=(asymptomatic OR afebrile OR symptomless)) AND ALL=(malaria) – 48 – Web of Science Core Collection](https://www.webofscience.com/wos/woscc/summary/5998a6ef-4579-49d3-aad7-59fe45408875-eb6635fa/relevance/1) | 47 |
| Citation search  07/10/2014 | Citations search | NA | 171 |
